# The Social Evil

**Published:** 1907-04

**Authors:** Edgar G. Ballenger

**Affiliations:** Lecturer on Genito-Urinary Dseases at the Atlanta School of Medicine, Atlanta, Ga.


### THE SOCIAL EVIL.*

By Edgar G. Ballenger, M. D.
Lecturer on Genito-Urinary Diseases at the Atlanta School of Medicine, Atlanta, Ga.

Mr. President and Gentlemen:

In complying with the request of the programme committee of your society to present a paper upon this subject, I do so with many misgivings. So varied are the views that prevail among conscientious workers along this line, and so bitter have been their controversies, that I approach it with considerable timidity, for I realize that it should be handled with much delicacy and perhaps more judgment and discretion than I possess. I will outline, however, plans that seem to me feasible, and if carried out would tend to improve the existing conditions.

*Read before the Georgia Sociological Society.

History is full of unsuccessful attempts to cope with this evil, and many of them have resulted in producing more harm than good. No effort can succeed that ignores three-fourths of the offenders, and the causes that produce prostitution. It would be just as rational to try to stamp out an epidemic of yellow fever by quarantining the women and allowing the men to run at large, as to expect to control the social evil by laws directed only against fallen woman while man with his excessive sexual activity is totally disregarded.

There is no one factor that has played a more important part in the history of the world than has lust and licentiousness, from Eve in the Garden of Eden, profligate Nero and the downfall of Rome, Louis the XV, his Du Barry and the French Revolution, down to our present day, when an eminent authority claims that the German Empire, in spite of her high civilization, unless she reforms, must inevitably follow those who have walked the downward path into the pit before her, on account of loose morals in regard to sexual matters. Many other powerful nations to-day are just as corrupt.

Directly in the wake of sensuality follow mental and physical depravity. Venereal diseases may be taken as an index of the extent of this condition and are surely its worst results.

Heredity and environment are powerful factors that are constantly at work. Lombroso and Madame Tarnowsky have shown that prostitutes may properly be classed as degenerates. Laws that allow promiscuous marriage of defectives can only expect to reap a harvest of the seed that are sown.

Unfavorable environment tends to degrade the normal and produce criminals of the degenerates. Alcoholism and venereal diseases account for the largest part of degeneracy and both are fostered by the social evil.

Irritative lesions in the urethral canal, prostate gland and the other genital organs, set up by chronic gonorrhoea, cause sexual hyperactivity which makes these patients difficult to control in their sexual hygiene. Thus we see the vicious circle that connects alcoholism, prostitution and venereal diseases.

"The prevention of disease is the keynote of human sympathy and the keynote of human endeavor." The prevention of innocent suffering is so thoroughly in accord with the best instincts of man that our most effective results in lessening the social evil will follow attempts for this avowed purpose.

The folly of the public punctilio and indifference concerning this subject can best be combatted by showing citizens the venereal peril of their sons and daughters, with the physical, mental and moral degradation that attends it. Public sentiment is the most powerful of all weapons with which to attack this or any other evil. To gain the influence of this factor we must show that our claims are not all fiction and our plans are not visionary and impracticable. Let us present in a forceful way facts that show:

That there is no greater scourge devastating every nation to-day than venereal diseases.

That gonorrhoea causes more innocent suffering among women than any other disease, bringing thousands to lives of invalidism and thousands of others to the operating table for major operations.

That it causes from 20 to 25 per cent. of all blindness in children.

That venereal diseases represent a most enormous direct monetary loss to the nation, yearly, and a still greater one indirectly through the lessened earning and producing capacity of its victims.

That they contribute large numbers to our insane asylums, hospitals and prisons.

That they are among the important factors producing race suicide.

That they are the direct cause, many times, of self destruction and divorces.

And yet, the public, on account of its prudishness and a sense of false modesty, continues to ignore the subject.

The prostitute, public and clandestine, male and female, is the fountain-head of these destructive diseases.

Many men carry gonorrhoea into their families without knowing the consequences. Morrow says there is more of it among virtuous wives than in professional prostitutes. Powerful motives, however, lead to its concealment.

LEGAL REGULATION.—Compulsory examination and treatment of prostitutes can only slightly mitigate this disease, even under the best circumstances. Every physician will admit that there are few conditions in medicine that are more difficult to diagnose than a latent gonorrhoea in a woman who will not co-operate, taking douches and urinating just before the examination. In such cases it is practically impossible to make a positive diagnosis. Even at this time if she were perfectly healthy, within a few hours after the issuance of such a recommendation, the germs may be implanted and flourish, spreading disease among patrons, who perhaps without the false assurance given by the certificate, would have refrained from intercourse. This and many other valid reasons make the legal regulation unsatisfactory and even dangerous. Laws to prohibit prostitution entirely simply drive them from their secluded quarters and make of the regular prostitute a clandestine one, who, in every respect is more dangerous, for in having her as our neighbor or in boarding houses, she is in position to do vastly more harm, by entrapping the weak or unwary, than if left undisturbed in a section of the city where to be seen implies the purpose of one's visit.

In order to reach the main points along which the fight against the social evil must be made, let us consider the various causes of the conditions to be encountered. This brings up many complex social problems, some of recent development, others that have existed from time immemorial.

EDUCATION.—Undoubtedly, ignorance, both public and private, is a cause of the indifference concerning the subject. The public does not realize the dangers to the State. The private individual does not know, in time, the far-reaching and sometimes disastrous results that are likely to follow promiscuous intercourse, even when the co-respondent appears to be perfectly clean and healthy.

The educational and sanitary crusade against tuberculosis has brought such excellent results that we should be encouraged in the campaign to lessen the social evil and social diseases. If the public could be aroused as to its urgent need, an important advance would be made. A law requiring practicing physicians to send in a record, every month, of the number of their patients (without names) having gonorrhoea and syphilis and the various complications and deaths that result therefrom, would at the expiration of a year, show such startling facts, when compared with the epidemic of yellow fever, small-pox and other reportable diseases, that even the most skeptical would be forced to the conclusion that our present method of inactivity should be abandoned.

The majority go into illicit sexual life knowing little of its dangers and pitfalls. They do not realize that in gratifying a passing desire they endanger their future health and happiness by a disease that may make it unsafe for them ever to marry. Or that if they do, a loathsome disease may be transferred to an unsuspecting wife or its stigma to an unborn babe. Few girls know that in yielding to a too ardent lover they may contract a serious disease, or that syphilis may be contracted by the kiss of a dissipated man. They all know vaguely of a "bad disease" that may result from such relations, but they are unaware of its frequency or the great likelihood of their contracting it. Young women, and especially working girls, from whom prostitutes are largely recruited, should be shown the logical and inevitable ending of such affairs.

Boys are sure to learn something about sexual matters and the more mysterious we make them the greater will be their curiosity. Unless we teach them facts, their ideas will be obtained from servants or older dissolute boys or young men who are posted on these matters and give the desired information in a boasting way, embellished with interesting experiences that tempt them to explore the fascinating and unknown realms of passion.

"Good education is better than bad. The bad is sure to be acquired, therefore let us substitute the good." The age at which this should be begun, according to Keyes, is from seven to twelve, "before the age of puberty, before the sexual idea in its emotional garb has entered the imagination." He thinks "they should be educated at home, in a kindergarten way, to a general knowledge of the idea that all life, of plant, of animal, of fish, of bird, comes from a previous life, and that as a rule it requires two previous lives before there can be any new life; that one of these lives is male life supplying something and the other is female supplying the rest; and this can be objectively illustrated through flowers and trees, and the instrumentality of insects in fertilizing the blossom and thus producing the seed and fruit. These facts can easily be brought into the child's comprehension without shock or shame. Then naturally as he grows older . . . he can be carried on through biological and zoological channels until he comes to look upon sex as being as natural to a plant as to an animal, and after that further instruction will be easy and may be extended to any desired limit." Keyes remarks that shyness on the part of the parent is quite natural, but that this is the best way for parents to be taught, and that it should be begun when the parent is a child.

SOCIAL PROBLEMS.—Poverty and destitution drive many women to prostitution as the only means of obtaining a livelihood, and especially is this true of those who have incurred social disgrace by the birth of an illegitimate child. The State should make greater efforts to reclaim these women before they become totally depraved and a menace to public welfare. Much to be commended are the establishments of homes, as the one recently started here in Atlanta by Mrs. E. M. Jackson, for young women who come to the city for employment and who have no home environment and are surrounded by the temptations of a homeless city life.

Love of fine clothing, jewelry and luxuries beyond their means, rather than lust or sexual desire, drive many along the road to ruin. They sell their virtue, health, morality and even their souls for a few showy baubles.

The close intermingling of the sexes in the various forms of industrial life is to be condemned. Working girls should receive more adequate compensation for their services and should be furnished suitable places of innocent amusement.

The demand for prostitutes should be lessened by encouraging men to remain continent and by assuring them that abstinence is perfectly compatible with health and sexual vigor. The number of men, if any, who are injured by refraining from sexual intercourse is infinitessimal when compared to the number who are diseased and rendered miserable, impotent neurasthenics by the venereal diseases they contract trying to exercise a function they fatuously believe must be used or lost. Nature takes ample care of the seminal secretion by emissions, but the quack and charlatan, by deceiving men as to their significance, make them the most remunerative of the complaints of their patients.

More rigid laws should be enacted to regulate the presentation of vulgar and suggestive plays, the publication of obscene literature and the plastering of bill-boards all over our cities with pictures that continually keep the mind upon things sexual and arouse amorous tendencies that more than counteract the good effect of a plea to remain continent. The nude and often lascivious "works of art" so constantly seen in saloons also act as a powerful suggestive factor, and at a time when the inhibition that would normally deter a man from such a step has been relaxed by alcohol, or when his sexual desire is stimulated by it. It is a well-known fact that the majority of men contract venereal diseases when drinking.

A glaring evil, present in every city to-day, is the impunity with which abortions are performed. The wholesome fear of becoming pregnant, knowing its resultant disgrace, would prevent many from taking the initial step, if it were not so easy to have a criminal abortion performed. I doubt if there is an adult person in Atlanta who does not know of establishments where this illegal and disgraceful work is done—some of them well known and upon our leading streets. And yet they are tacitly ignored. Why? Because no one cares to gain the notoriety that would result from an effort to prosecute them. An organized society can do this, however, without undesirable publicity for any of its members.

One of the perplexing problems that confronts us is the fact that houses of ill fame are largely supported by old men and married men. The thought is a revolting one, but unfortunately too true.

Lack of affinity and incompatibility in married life is a cause of prostitution that is beyond our reach.

In all I have said, nothing could avail so much as for men to curb their desires and exercise their faculty of self-restraint, but they will not.

Gentlemen, we should organize a society here in Atlanta to mitigate, at least, this evil and to co-operate with similar organizations that are being formed in all large cities for the education of the public as to these matters, and to combat the foolish "cultivation of ignorance" concerning such an important and far-reaching subject.

We must constantly bear in mind, though, that the prostitute herself is largely a victim of environment, poverty and degeneracy, and consequently all our animosity should not be hurled at her, for she is truly "more to be pitied than censured."

To attain results, we must attack the factors in our social system that produced her and make such a life profitable, although a life disgraceful and short, and let us try to remove the cause rather than control the effect.

1014-15 Century Building.

---

Lieutenant James Carroll, curator of the Army Medical Museum, the officer who submitted to a test upon himself in order to determine whether or not the germs of yellow fever were carried by mosquitoes, is to receive a deserved promotion. The House of Representatives has passed a bill authorizing the President to appoint him a major in recognition of his bravery and on account of the invalidism that has followed yellow fever contracted in this manner.

---

The amalgamation of the medical societies of London has at last been effected, under the name of the "Royal Society of Medicine." The names "Royal Academy of Medicine" and "Imperial Academy of Medicine" were rejected.

